# Identify diabetic retinopathy-related clinical concepts and their attributes using transformer-based natural language processing methods

**DOI:** 10.1186/s12911-022-01996-2

**Published:** 2022-09-27

**Authors:** Zehao Yu, Xi Yang, Gianna L. Sweeting, Yinghan Ma, Skylar E. Stolte, Ruogu Fang, Yonghui Wu

**Affiliations:** 1 Department of Health Outcomes and Biomedical Informatics, College of Medicine, University of Florida, Gainesville, FL USA; 2 Department of Biomedical Engineering, College of Engineering, University of Florida, Gainesville, FL USA

**Keywords:** Diabetic retinopathy, Natural language processing, Named entity recognition, Deep learning, Relation extraction

## Abstract

**Background:**

Diabetic retinopathy (DR) is a leading cause of blindness in American adults. If detected, DR can be treated to prevent further damage causing blindness. There is an increasing interest in developing artificial intelligence (AI) technologies to help detect DR using electronic health records. The lesion-related information documented in fundus image reports is a valuable resource that could help diagnoses of DR in clinical decision support systems. However, most studies for AI-based DR diagnoses are mainly based on medical images; there is limited studies to explore the lesion-related information captured in the free text image reports.

**Methods:**

In this study, we examined two state-of-the-art transformer-based natural language processing (NLP) models, including BERT and RoBERTa, compared them with a recurrent neural network implemented using Long short-term memory (LSTM) to extract DR-related concepts from clinical narratives. We identified four different categories of DR-related clinical concepts including lesions, eye parts, laterality, and severity, developed annotation guidelines, annotated a DR-corpus of 536 image reports, and developed transformer-based NLP models for clinical concept extraction and relation extraction. We also examined the relation extraction under two settings including ‘gold-standard’ setting—where gold-standard concepts were used–and end-to-end setting.

**Results:**

For concept extraction, the BERT model pretrained with the MIMIC III dataset achieve the best performance (0.9503 and 0.9645 for strict/lenient evaluation). For relation extraction, BERT model pretrained using general English text achieved the best strict/lenient F1-score of 0.9316. The end-to-end system, BERT_general_e2e, achieved the best strict/lenient F1-score of 0.8578 and 0.8881, respectively. Another end-to-end system based on the RoBERTa architecture, RoBERTa_general_e2e, also achieved the same performance as BERT_general_e2e in strict scores.

**Conclusions:**

This study demonstrated the efficiency of transformer-based NLP models for clinical concept extraction and relation extraction. Our results show that it’s necessary to pretrain transformer models using clinical text to optimize the performance for clinical concept extraction. Whereas, for relation extraction, transformers pretrained using general English text perform better.

## Background

Diabetic Retinopathy (DR), a common complication of diabetes, is the leading cause of blindness in American adults and the fastest growing disease threatening nearly 415 million diabetic patients worldwide [1, 2]. This disease may cause no symptoms or only mild vision problems but eventually, can cause blindness. With professional eye imaging devices such as fundus cameras or Optical Coherence Tomography (OCT) scanners, most vision-threatening diseases can be curable if detected [3]. Therefore, detection is very important for effective treatment of DR. Recent development of Artificial Intelligence (AI) technology greatly improved the autonomous DR diagnosis systems including the referral system from Google AI and the FDA-approved iDx-DR, which make the detection of vision-threatening diseases from a low-cost mobile camera available.

Electronic Health Records (EHR) have been increasingly implemented at US hospitals. Huge amounts of longitudinal patient data have been accumulated and are available electronically in structured tables, narrative text, and images. There is an increasing need for multimodal learning methods to link different data sources for clinical and translational studies. Recent emerging AI technologies, especially deep learning (DL) algorithms, have greatly improved the performance of automated vision-disease diagnoses systems based on EHR data. These AI systems for vision-disease diagnoses are usually developed using supervised machine learning models with medical images. The supervised machine learning models require annotated images, where the annotator have to manually label the region with lesions from images. In fact, the physicians have reviewed these medical images and documented detailed diagnosis, symptoms, and other critical observations in image reports, which could be a valuable resource to help annotators label images or serve as independent text features for lesion detection from medical images. There are increasing numbers of clinical studies utilizing clinical narratives [4–7]. As the emergence of precision medicine, more and more studies look into clinical narratives to generate a more complete picture of patients to better assess health outcomes [8].

Natural language processing (NLP) is the key technology to extract patient information from clinical narratives to support various downstream clinical studies. Many NLP methods and systems have been developed to extract various types of information from clinical narratives. The clinical NLP community has organized a number of open challenges to advance information extraction from clinical narratives. Most state-of-the-art NLP methods for information extraction are based on supervised machine learning methods. The supervised machine learning models approach the information extraction as a two-stage pipeline, which typically include a clinical concept extraction (or named entity recognition [NER]) module to identify critical concepts (e.g., diseases, medications) and a relation extraction module to link attributes (e.g., negations, disease severity) to the concepts. For concept extraction, a number of NLP models have been developed to first identify clinical concepts and their attributes and then classify them into predefined semantic categories (e.g., diseases, medications). Relation extraction aims to establish semantic connections between extracted concepts and their attributes. Recently, transformer-based NLP models, built solely with a self-attention mechanism, outperformed other models and became state-of-the-art solution for information extraction from clinical narratives. For example, Peng et al. [9] proposed a BERT-based model for relation extraction; Dat et al. [7] proposed an end-to-end NLP model for relation and entity recognition in general English. However, the clinical text data is rarely used for developing AI systems for diagnosing DR and most studies on DR focused on medical images and structured EHRs. For example, Wong et al. [10] proposed a three-layer feed-forward neural network to detect the microaneurysms and hemorrhage from medical images; Imani et al. [11] applied morphological component analysis to detect the exudation and blood vessel; Sun et al. [12] proposed a machine learning model to diagnose potential DR in patients using structured EHR data. There are studies exploring clinical narratives for text classification and computable phenotyping of DR. For example, Yang et al. [4] examined deep learning models to identify progress notes related to diabetes; Jin et al. [13] developed an NLP System to detect hypoglycemia-related events; Wu et al. [14] proposed a rule-based NLP system to help identify DR patients using clinical narratives. To the best of our knowledge, there are limited studies applying state-of-the-art transformer-based NLP models to extract DR-related clinical concepts from clinical narratives.

In this study, we identified patients diagnosed with DR at the University of Florida (UF) Health and collected their image reports, developed annotation guidelines and annotated a corpus for DR-related concept extraction, developed transformer-based NLP methods to extract DR-related clinical concepts that could help lesion detection from medical images. We systematically examined two state-of-the-art transformer-based NLP models for DR-related concept extraction and relation extraction from fundus image reports. We also developed end-to-end systems to detect DR-related concepts as well as their attributes in a unified system.

## Methods

### Data sets

We identified 155 patients diagnosed with diabetic retinopathy and collected a total number of 536 fundus image reports from them at the University of Florida (UF) Health. Then, we developed initial annotation guidelines through a collaboration of clinicians specialized in DR treatment, computer image experts (RF, SES, GLS), and NLP experts (YW, XY, ZY). Then, we recruited two annotators (YM, GLS) and conducted training sessions to help annotators get familiar with guidelines. We further improved the initial guidelines using several training sessions. After the annotators achieved a good inter-annotator agreement score calculated using Cohen’s Kappa [15] we conducted 3 rounds of annotation and finished the annotation of 536 notes. The first round (40 reports) was double-annotated to assess inter-annotator agreement. After each round of annotation, we discussed the discrepancies in group meetings among annotators, physicians, and researchers, updated the annotation guidelines, and revised the annotations as needed. This study was approved by UF Institutional Review Board (IRB201801358).

### DR-related concepts

There are many DR-related clinical concepts documented in the image reports such as diagnoses, treatments, and medications. As our goal is to extract DR concepts that can potentially help lesion detection from medical images, we identified five different categories of concepts, including lesions, eye parts, laterality, severity, and negation. By definition, a lesion is a region in an organ or tissue which has suffered damage through injury or disease. In this study, we are particularly interested in lesions only associated with diabetic retinopathy (lesion occurred within the eye). Lesions that occurred in other organs were not be annotated. We also referred to the existing vocabulary of lesions [16, 17], and domain experts’ knowledge to develop the annotation guidelines. When annotating a lesion, we asked annotators to annotate the associated attributes including eye-part, laterality, severity, and negation. The annotators were asked to first identify the lesions and their attributes, and then link the attributes to the corresponding lesions using three relations including ‘located, ‘laterality-lesion’, ‘severity-lesion’.

### Annotation tool

**Fig. 1:**
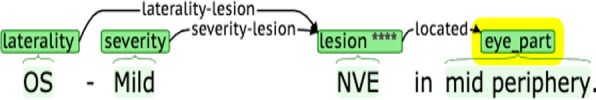
An example of brat annotation for diabetic retinopathy (DR)

We used the brat rapid annotation tool [18] for annotation. Fig. 1 shows an example of a DR-related lesion concept and the identified eye part, laterality, and severity.

### NLP methods

We adopted a standard two-stage NLP pipeline, including a clinical concept extraction module to detect DR-related concepts and their attributes and a relation extraction module to link the attributes to the corresponding concepts. Many studies have examined rule-based and machine learning-based methods for information extraction from clinical narratives and showed that the machine learning-based methods often have better performance and generalizability. When applying rule-based NLP systems to a new dataset, researchers often have to customize the rules according to the new patterns and documenting styles. [19] Therefore, we focused on machine learning-based methods based on state-of-the art deep learning models. For concept extraction, we explored two state-of-the-art transformer-based NLP methods, including Bidirectional Encoder Representations from Transformers (BERT) [20] and Robustly optimized BERT approach (RoBERTa) [21] as they showed better performance in our previous study [22]. BERT is a bidirectional transformer-based NLP model based on masked language modeling (MLM) and uses next-sentence prediction (NSP) to learn representations from text. RoBERTa is a transformer-based language model shared the same architecture as BERT but pretrained with a dynamic MLM where masking patterns were generated during the training with different random seeds. We compared BERT and RoBERTa with a Long short-term memory (LSTM) model with CRF layer as a baseline, which was implemented using Tensorflow in our previous study [23]. For transformer-based NLP models, we used the implementations from our clinical transformer package [22] based on the transformer architectures from the HuggingFace [24] in PyTorch [25]. For relation extraction task, we used the implementations from our clinical relation extraction with transformer package [26] based on the transformer architectures. Our relation extraction pipeline consists of two steps including (1) identifying pairs of concepts that potentially have a relation, and (2) classifying the relation categories using machine learning classifiers. We explored two state-of-the-art transformer-based NLP methods, including BERT and RoBERTa, as they achieved good performance in our previous study. As shown in Fig. 1, most relations between concepts occurred in the same sentence. Thus, we implemented heuristic rules to only consider two concepts occurring in the same sentence as a candidate pair for relation classification. More specifically, the heuristic rule will generate a candidate pair between a ‘lesion’ concept and an ‘eye part’ concept as there is a relation defined between them; but it won’t generate a candidate pair between a ‘severity’ concept and an ‘eye part’ concept as there is no relation defined in between. Then, we applied a binary classification strategy to determine whether the candidate pair has a relation (positive) or not (negative).

For the LSTM-CRFs model, following previous study on clinical concept extraction [27], we explored general models (LSTM_general) trained using English corpus using fastText [28] and compared the general models with clinical models (LSTM_clinic) trained using clinical notes from the Medical Information Mart for Intensive Care III (MIMIC-III) with the fastText algorithm. For Transformer models, we used the ‘base’ setting in this study. Following our previous studies [22, 29, 30] on clinical transformers, we also examined pre-trained transformers from general English corpus (denoted as ‘_general’, e.g., ‘BERT_general’) and clinical transformers pre-trained using clinical notes from the MIMIC-III database [31] (denoted as ‘_mimic’, e.g., ‘BERT_mimic’). We applied the default tokenizer in each model (e.g. wordpiece[32] in BERT and Byte-Pair Encoding [33] in RoBERTa) and adopted the default parameters optimized in our clinical transformer package and clinical relation extraction with transformer package [22, 26]. We adopted a widely used negation detection algorithm, NegEx [34], to handle the negations. To improve the accuracy of negation detection, we customized the NegEx program using negation words identified from the training set. For relation extraction, we examined the transformer-based models under two settings, including (1) a pure relation extraction task where we assume that all concepts and their attributes are known and we only focus on how to identify the candidate pairs and classifier them into predefined categories, and (2) an end-to-end task to first identify the concepts and their attributes and then identify the relations (denoted as ‘e2e’). For the end-to-end system, we applied the best model in concept extraction (BERT_mimic model) to generate candidate pairs and examined transformer models for relation classification. More specifically, we utilized transformers to learn a sentence-level representation for the input sentence and two concept-level representations (for the two concepts in a candidate pair) and then concatenated them as input for a soft-max layer for classification.

### Evaluation

We evaluated annotation agreement using Cohen’s Kappa, κ, coefficient, where higher κ denotes annotator agreement. We used both strict (i.e., the beginning and end boundaries of a concept have to be exactly the same with gold-standard annotation) and lenient precision, recall, and F1-score to evaluate our NLP systems for concept extraction. Precision is defined as (the number of predicted concepts correctly identified by the NLP system) / (total number of concepts identified by NLP); recall is defined as (the number of predicted concepts correctly identified by the NLP system) / (total number of concepts annotated by experts); F1-score is defined as “(2*precision*recall)/(precision + recall)”. We used the micro average to calculate the overall score. We used accuracy to evaluate negation detection, which is defined as (the number of concepts with negations correctly identified) / (total number of concepts annotated by experts).

## Results

Two annotators annotated a total number of 4,782 DR-related concepts from 536 reports. We calculated the inter-annotator agreement score using 40 overlapped reports. For concept extraction, two annotators achieved an F1-score of 0.8021(in concept-level) and a token-level kappa score of 0.74. For relation annotation, two annotators achieved an F1-score of 0.7542. We randomly divided the dataset into a training set and a test set with an 8:2 ratio. Table 1 shows the distribution of notes and DR-related concepts in the training and test set. We used the training set to develop transformer-based NLP models and used the test set for evaluation.

**Table 1:** Concepts distributions for training and test

	Training set	Test set	Total	Example concept
Total notes	391	145	536	
Lesion	2,383	896	3,279	‘hemorrhage’
Laterality	1,280	485	1,765	‘right eye’
Severity	579	249	828	‘mild’
Eye part	45	17	62	‘foveal’
Total concepts	4,287	1,647	5,934	

Table 2 shows the number of negated/non-negated concepts in the training and test set. Without customization, the original NegEx algorithm achieved an accuracy of 0.62. After customizing the NegEx algorithm using the training set, the customized algorithm achieved an accuracy of 0.9265.

**Table 2:** Negation attributes distributions for training and test

	Training set	Test set	Total
Total notes	391	145	536
Non-negated_lesion	2,057	747	2,804
Negated_lesion	416	149	901

Table 3 compares six different NLP methods in extracting DR-related concepts from fundus image reports. All six methods performed well for concept extraction. The two transformer-based models outperformed the baseline LSTM model. Among four transformer-based models, the models pretrained using clinical notes from the MIMIC-III database outperformed their corresponding models pretrained using general English corpora. Among the two transformer-based NLP models trained using clinical text, the BERT_mimic model achieved the best strict/lenient F1-score of 0.9503 and 0.9645 on the test set, respectively. Table 4 shows the detailed performance for each of the four DR-related categories for the best NER model based on BERT. The BERT_mimic achieved lenient F1-scores over 0.95 for lesion, severity, and laterality, where the performance for detecting lesion is the best, which has a strict/lenient F1-score of 0.9565 and 0.9750, respectively; the performance for eye part category is relatively low with F1-score of 0.75.

**Table 3:** Performance comparison for concept extraction

	Strict			Lenient		
	Precision	Recall	F1 score	Precision	Recall	F1 score
LSTM_general	0.9492	0.9186	0.9337	0.9630	0.9320	0.9472
LSTM_mimic	0.9464	0.8682	0.9056	0.9609	0.8810	0.9192
BERT_general	0.8885	0.9575	0.9217	0.9067	0.9739	0.9391
BERT_mimic	0.9486	0.952	**0.9503**	0.9642	0.9648	**0.9645**
RoBERTa_general	0.9248	0.9636	0.9438	0.9353	0.9739	0.9542
RoBERTa_mimic	0.9391	0.9551	0.947	0.9498	0.9654	0.9575

^*^Best F1 scores are highlighted in bold

**Table 4:** Detailed performance for each concept category for BERT_mimic

	Strict			Lenient		
	Precision	Recall	F1 score	Precision	Recall	F1 score
Lesion	0.9555	0.9576	0.9565	0.9776	0.9743	0.976
Severity	0.9627	0.9317	0.9469	0.9668	0.9357	0.951
Eye part	0.8	0.7059	0.75	0.8	0.7059	0.75
Laterality	0.9339	0.9608	0.9472	0.9439	0.9711	0.9573
Overall	0.9486	0.952	0.9503	0.9642	0.9648	0.9645

Table 5 compares the two transformer-based NLP models for relation extraction under a gold-standard concept setting and an end-to-end setting. In the end-to-end systems, we applied the the best model for concept extraction—the BERT_mimic model. Using gold-standard concepts, the BERT_general achieved the best lenient/strict F1-scores of 0.9316. For the end-to-end setting, both BERT_general model and RoBERTa_general model achieved the best performance of 0.8578 using the strict evaluation. The BERT_general model achieved the best lenient F1-scores of 0.8881 under the end-to-end setting.

**Table 5:** Performance comparison for relation extraction models

Settings	NLP Models	Strict			Lenient		
		Precision	Recall	F1 score	Precision	Recall	F1 score
Use gold-standard concepts	BERT_general	0.9199	0.9437	**0.9316**	0.9199	0.9437	**0.9316**
	RoBERTa_general	0.9024	**0.9574**	0.9291	0.9024	**0.9574**	0.9291
	BERT_MIMIC	**0.9254**	0.9254	0.9254	**0.9254**	0.9254	0.9254
	RoBERTa_MIMIC	0.9147	0.9467	0.9304	0.9147	0.9467	0.9304
End-to-end	BERT_general_e2e	**0.8397**	0.8767	**0.8578**	**0.8712**	0.9056	**0.8881**
	RoBERTa_general_e2e	0.8274	**0.8904**	**0.8578**	0.8565	**0.9178**	0.8861
	BERT_MIMIC_e2e	0.8282	0.8584	0.843	0.8584	0.8858	0.8719
	RoBERTa_MIMIC_e2e	0.8362	0.8782	0.8567	0.8688	0.9072	0.8876

^*^Best precision, recall, and F1 are highlighted in bold. The strict and lenient scores are identical for the ‘gold-standard’ settings as the gold-standard annotation for concepts and attributes were used

## Conclusions

Identify DR-related concepts is a critical step to leverage clinical narratives for lesion detection from the medical image. In this study, we developed annotation guidelines to annotate DR-related concepts from fundus image reports, annotated a corpus of 536 image reports with four categories of clinical concepts and two state-of-the-art transformer-based NLP models for detecting DR-related concepts and relations. For negation detection, we customized an existing negation detection algorithm, NegEx, using the training set and improved the accuracy from 0.62 to 0.9265, indicating it’s necessary to customize the rule-based negation detection algorithms using local datasets. For concept extraction, three out of four transformer-based models achieved better performance than the baseline model, except for the BERT_general model. The BERT model pretrained with the MIMIC III dataset achieved the best lenient F1-score of 0.9645. From Table 3, we noticed that the best model BERT_mimic achieved a good performance for lesion, severity, and laterality concepts, whereas, the performance for the eye part concept is relatively lower. One potential reason for the low performance for eye part concepts is there is limited number of concepts annotated compared with other categories. The transformer models pretrained using clinical text from the MIMIC III outperformed transformer models pretrained using general English corpora, which is consistent with findings reported in work [35, 36]. Similar to other clinical concept extraction tasks, fine-tuning the pre-trained transformers can further help improve the performance of extracting DR-related concepts.

We further link the severity, laterality, and eye part concepts to the corresponding lesion concept using relation extraction. The BERT_general model achieved the best strict/lenient scores of 0.8578 and 0.8881 for both settings, respectively. The RoBERTa_general also achieved the same performance as BERT_general in the strict evaluation score as a tie. Overall, the performance difference between the two transformer-based models in the end-to-end setting is not that significant with the setting using gold-standard concepts. It’s not surprising to see that the performances for end-to-end systems are lower (~ 8% lower in strict evaluation and ~ 5% lower in lenient evaluation) than pure relation extraction using gold-standard concepts.

## Discussion

This study has limitations. The dataset we developed in this study is relatively clean without complex situations for relation extraction. For example, most of the relations are located at the same sentence. As the ultimate goal is to leverage the clinical narratives to help lesion detection from medical images, we plan to develop multimodal visual-text learning models to combine clinical text and medical images for early detection of DR in future studies.

## Data Availability

The datasets generated and/or analyzed during the current study are not publicly available due to patient privacy information but are available from the corresponding author on reasonable request.

## Abbreviations

DR: Diabetic retinopathy
NLP: Natural language processing
NER: Named entity recognition
EHR: Electronic health records
DL: Deep learning
LSTM: Long-short term memory
UF: University of Florida
OCT: Optical coherence tomography
AI: Artificial intelligence
BERT: Bidirectional encoder representations from transformers
RoBERTa: Robustly optimized BERT approach
MLM: Masked language modeling
NSP: Next-sentence prediction
MIMIC-III: Medical information mart for intensive care III

## References

[1] Bourne RRA, Stevens GA, White RA, Smith JL, Flaxman SR, Price H (2013). Causes of vision loss worldwide, 1990–2010: a systematic analysis. Lancet Glob Health.

[2] Mohamed Q, Gillies MC, Wong TY (2007). Management of diabetic retinopathy: a systematic review. JAMA.

[3] Gao Z, Li J, Guo J, Chen Y, Yi Z, Zhong J (2019). Diagnosis of diabetic retinopathy using deep neural networks. IEEE Access.

[4] Yang B, Wright A. Development of deep learning algorithms to categorize free-text notes pertaining to diabetes: convolution neural networks achieve higher accuracy than support vector machines. arXiv:1809.05814. 2018

[5] Bucher BT, Shi J, Pettit RJ, Ferraro J, Chapman WW, Gundlapalli A (2020). Determination of marital status of patients from structured and unstructured electronic healthcare data. AMIA Annu Symp Proc.

[6] Stubbs A, Filannino M, Soysal E, Henry S, Uzuner Ö (2019). Cohort selection for clinical trials: n2c2 2018 shared task track 1. J Am Med Inform Assoc.

[7] Nguyen DQ, Verspoor K. End-to-end neural relation extraction using deep biaffine attention. arXiv:1812.11275. 2019;11437:729–38.

[8] Khalifa A, Meystre S (2015). Adapting existing natural language processing resources for cardiovascular risk factors identification in clinical notes. J Biomed Inform.

[9] Shi P, Lin J. Simple BERT models for relation extraction and semantic role labeling. arXiv:1904.05255. 2019

[10] Yun WL, Rajendra Acharya U, Venkatesh YV, Chee C, Min LC, Ng EYK (2008). Identification of different stages of diabetic retinopathy using retinal optical images. Inf Sci.

[11] Imani E, Pourreza H-R, Banaee T (2015). Fully automated diabetic retinopathy screening using morphological component analysis. Comput Med Imaging Graph.

[12] Sun Y, Zhang D (2019). Diagnosis and analysis of diabetic retinopathy based on electronic health records. IEEE Access.

[13] Jin Y, Li F, Yu H. HYPE: a high performing NLP system for automatically detecting hypoglycemia events from electronic health record notes. arXiv:1811.11945. 2018

[14] Wu H, Wei Y, Shang Y, Shi W, Wang L, Li J (2018). iT2DMS: a standard-based diabetic disease data repository and its pilot experiment on diabetic retinopathy phenotyping and examination results integration. J Med Syst.

[15] McHugh ML (2012). Interrater reliability: the kappa statistic. Biochem Med.

[16] Duh EJ, Sun JK, Stitt AW (2017). Diabetic retinopathy: current understanding, mechanisms, and treatment strategies. JCI Insight.

[17] Wang W, Lo ACY (2018). Diabetic retinopathy: pathophysiology and treatments. Int J Mol Sci.

[18] Stenetorp P, Pyysalo S, Topić G, Ohta T, Ananiadou S, Tsujii J. Brat: a web-based tool for NLP-assisted text annotation. In: Proceedings of the demonstrations at the 13th conference of the European chapter of the association for computational linguistics. Avignon, France: Association for Computational Linguistics; 2012. p. 102–7.

[19] Gehrmann S, Dernoncourt F, Li Y, Carlson ET, Wu JT, Welt J, et al. Comparing rule-based and deep learning models for patient phenotyping. arXiv:1703.08705. 2017. 10.1371/journal.pone.0192360 PMC5813927 29447188

[20] Devlin J, Chang M-W, Lee K, Toutanova K. BERT: Pre-training of deep bidirectional transformers for language understanding. arXiv:1810.04805. 2019

[21] Liu Y, Ott M, Goyal N, Du J, Joshi M, Chen D, et al. RoBERTa: a robustly optimized BERT pretraining approach. arXiv:1907.11692. 2019

[22] Yang X, Bian J, Hogan WR, Wu Y (2020). Clinical concept extraction using transformers. J Am Med Inform Assoc.

[23] Wu Y, Jiang M, Xu J, Zhi D, Xu H (2018). Clinical named entity recognition using deep learning models. AMIA Annu Symp Proc.

[24] Wolf T, Debut L, Sanh V, Chaumond J, Delangue C, Moi A, et al. HuggingFace’s transformers: state-of-the-art natural language processing. arXiv:1910.03771.. 2020

[25] Paszke A, Gross S, Massa F, Lerer A, Bradbury J, Chanan G, et al. PyTorch: An imperative style, high-performance deep learning library. arXiv:1912.01703. 2019

[26] Yang X, Yu Z, Guo Y, Bian J, Wu Y. Clinical relation extraction using transformer-based models. arXiv:2107.08957. 2021

[27] Yang X, Lyu T, Li Q, Lee C-Y, Bian J, Hogan WR (2019). A study of deep learning methods for de-identification of clinical notes in cross-institute settings. BMC Med Inform Decis Mak.

[28] Joulin A, Grave E, Bojanowski P, Douze M, Jégou H, Mikolov T. FastText.zip: compressing text classification models.arXiv:1612.03651. 2016

[29] Yang X, Zhang H, He X, Bian J, Wu Y (2020). Extracting family history of patients from clinical narratives: exploring an end-to-end solution with deep learning models. JMIR Med Inform.

[30] Yang X, He X, Zhang H, Ma Y, Bian J, Wu Y (2020). Measurement of semantic textual similarity in clinical texts: comparison of transformer-based models. JMIR Med Inform.

[31] Johnson AEW, Pollard TJ, Shen L, Lehman LH, Feng M, Ghassemi M (2016). MIMIC-III, a freely accessible critical care database. Sci Data.

[32] Schuster M, Nakajima K. Japanese and Korean voice search. In: 2012 IEEE international conference on acoustics, speech and signal processing (ICASSP). 2012. p. 5149–52

[33] Sennrich R, Haddow B, Birch A. neural machine translation of rare words with subword units. arXiv:1508.07909. 2016

[34] Chapman WW, Bridewell W, Hanbury P, Cooper GF, Buchanan BG (2001). A simple algorithm for identifying negated findings and diseases in discharge summaries. J Biomed Inform.

[35] Ji Z, Wei Q, Xu H (2020). BERT-based ranking for biomedical entity normalization. AMIA Jt Summits Transl Sci Proc.

[36] He Y, Zhu Z, Zhang Y, Chen Q, Caverlee J. Infusing disease knowledge into BERT for health question answering, medical inference and disease name recognition. arXiv: 2010.03746. 2020.

